# Induction of B7-H1 expression by human cytomegalovirus in extravillous cytotrophoblast cells and role of MAPK pathway

**DOI:** 10.12669/pjms.305.5144

**Published:** 2014

**Authors:** Wenrong Gong, Jianhua Zhao, Zhen Chen, Lin Lei, Lihua Luo, Xuehong Zhao, Hui Xing, Suhua Chen, Qisheng Tu

**Affiliations:** 1 Wenrong Gong, MD, Center for Molecular Medicine of Medical College/ Central Laboratory of the Affiliated Hospital, Hubei University of Arts and Science, Xiang Yang, Hubei, China 441053.; 2 Jianhua Zhao, MD, Department of Obstetrics and Gynaecology, Tongji Hospital, Tongji Medical College of Huazhong University of Science and Technology, Wuhan, 430030, China.; 3 Zhen Chen, MD, Department of Ophthalmology, Renmin Hospital, Wuhan University, Wuhan 430060, China.; 4 Lin Lei, MD,Department of Oncology, The Affiliated Hospital of Hubei University of Arts and Science, Xiang Yang, Hubei, China 441021.; 5 Lihua Luo, MD, Center for Molecular Medicine of Medical College/ Central Laboratory of the Affiliated Hospital, Hubei University of Arts and Science, Xiang Yang, Hubei, China 441053.; 6 Xuehong Zhao, MD, Center for Molecular Medicine of Medical College/ Central Laboratory of the Affiliated Hospital, Hubei University of Arts and Science, Xiang Yang, Hubei, China 441053.; 7 Hui Xing, MD, Department of Obstetrics and Gynaecology, The Affiliated Hospital of Hubei University of Arts and Science, Xiang Yang, Hubei, China 441021.; 8 Suhua Chen, MD, Department of Obstetrics and Gynaecology, Tongji Hospital, Tongji Medical College of Huazhong University of Science and Technology, Wuhan, 430030, China.; 9 Qisheng Tu, MD, Oral Biology, Tufts University School of Dental Medicine, 1 Kneeland Street, Boston, MA 02111, USA.

**Keywords:** Cytomegalovirus, Cytotrophoblast, B7-H1, MAPK

## Abstract

***Objective: ***This paper is aimed at to evaluate B7-H1 expression as induced by human cytomegalovirus (HCMV) in extravillous cytotrophoblast cell line HPT-8 and possible underlying mechanism.

***Method: ***Real time PCR and flow cytometry were used to determine B7-H1 mRNA and protein before and after HCMV infection in HPT-8 cells. Western blot analysis was used to determine the level of MAPK phosphorylation in HPT-8 cell lines infected with HCMV.

***Results: ***100TCID50 was found to be the most effective dose, capable of stimulating B7-H1 mRNA and protein expression in HPT-8 cells. When empty control group was considered to have a B7-H1 mRNA value of 1, B7-H1 mRNA was 4.32 in 100TCID50 group. In flow cytometry study, mean ﬂuorescence intensity (MFI) of 100TCID50 group was 16.14, while empty control group was 1.34. Both mRNA and protein expression were found to be significantly increased (P<0.05) in 100TCID50 group compared to empty control group. The result of Western blot analysis showed increase in B7-H1 expression caused by the extracellular signaling that was related to ERK activation and the ERK inhibitor U0126 was found to reverse this increase.

***Conclusion: ***HCMV upregulates B7-H1 expression in human extravillous cytotrophoblast cell line HPT-8, which is related to MAPK activation. Our result would be helpful in finding better therapies against intrauterine HCMV infection.

## INTRODUCTION

Maternal immunological tolerance of the fetal placenta involves the recognition of foreign antigens, especially, the paternally-inherited fetal antigens in such a way that does not induce an anti-fetal maternal immune response.^1^ Trophoblast cells located between the fetus and mother, can bias the maternal immune system toward immunological acceptance of fetal antigens. The mechanisms by which lymphocyte tolerance toward trophoblast cells have not been fully elucidated. However, different evidences have suggested that immune costimulator B7 family plays an important role in this process.

B7s providing second signals to immunological cells are key regulators of the adaptive immune response. Individual members of the B7 family can be both positive and negative regulators of the immune response depending on their specific counterreceptors.^   2 ^ B7-H1(PD-L1) is a negative regulator of the immune response in this family, which is important for formation and maintenance of maternal immunological tolerance of the fetal placenta.^2^ It is a surface-bound ligand and it binds to the lymphocyte-expressed receptor, PD-1. This interaction inhibits antigen-specific activation and cytokine production by T cells. In the human placenta, B7-H1 proteins are expressed selectively on trophoblast cells, including villous and extravillous cytotrophoblast cells, and syncytiotrophoblast cells. ^3^ Blockage of maternally-derived B7-H1 has been found to result in rejection of allogeneic fetuses in mice.^      3 ^^,^^      4 ^ It has also been shown that persistent expression of PD-1 on T cells contributes to chronic viral infections due to its inhibition of immune response.^      5 ^ In summary, these studies have offered strong evidence that this pathway plays a role in tolerance to both self and foreign antigens.

HCMV is an important reason for intrauterine infection which is one of the most common causes of microcephaly, sensorineural deafness, and retardation of cognitive abilities and intrauterine growth. In this paper, we report the influence of HCMV infection on the expression of immune co-suppressive molecule B7-H1. We also checked signaling protein’s phosphorylation after HCMV infection. Our results suggested HCMV was a strong inducer of B7-H1 expression in extravillous cytotrophoblast cell line HPT-8, this might explain why HCMV was a common factor for intrauterine infection and might provide clues for future anti- HCMV intrauterine infection strategy.

## METHODS

***Reagents: ***Extravillous cytotrophoblast cell line HPT-8, Human embryonic lung fibroblasts (HEL) and HCMV AD169 Standard strain were purchased from American Type Culture Collection (ATCC; Manassas, VA, U.S.). Cell culture reagents were all obtained from Gibco (Carlsbad, CA, U.S.). TRIzol Reagent, OPTI-MEM and Lipofectamine 2000 were from Invitrogen (Carlsbad, CA, U.S.). Anti-human B7-H1 antibody (clone MIH1) were from eBioscience (San Diego, CA, U.S.). Horseradish peroxidase (HRP)-conjugated goat anti-mouse and anti-rabbit IgG secondary antibody were obtained from Jackson Immunoresearch Laboratories Inc. (West Grove, PA, U.S.). All other antibodies were obtained from Cell Signaling Technology (Beverly, MA, U.S.). JNK inhibitor-SP600125, ERK inhibitor-U0126, P38 inhibitor-SB203580 were all from Calbiochem (U.S). Reverse transcriptase was obtained from Fermentas. SYBR Green PCR master mix for real time PCR was from Applied Biosystems.

***Cell culture: ***Both HEL and HPT-8 were cultured in DMEM with 10% fetal bovine serum, 100 U/ml penicillin, 100 µg/ml streptomycin and 1% nonessential amino acid. The cultures were maintained in an incubator at 37°C in 5% CO2 /95% air atmosphere.

***Determination of virulence: ***HEL cells were adjusted to 2×10^5^/ml with culture medium, 100 μl of which was added to each well of 96-well plate. After 48 hours of incubation, the medium was removed and different titers of virus (10^-1^-10^-7^) were added to each well in a volume of 100 μl. The experiment was repeated 4 times. Cell growth conditions were then observed every day for 7 days. A 50% tissue culture infective dose (TCID50) was calculated using the Reed-Muench method.

***Reverse transcription and*** ***real time PCR: ***TRIzol-Reagents were used to extract total cellular RNA. Reverse transcription (RT) was performed using 1.5 µg of total RNA and 0.2 µg of random hexamer primers according to the manufacturer’s protocol. 

Real time PCR was conducted in triplicate on an Applied Biosystems 7500 real-time cycler to determine the level of B7-H1 mRNA. The primers used for 18S (NCBI: NR_046235.1) were 5′- GGACACGGACAGGATTGACA -3′ and 5′- ACCCACGGAATCGAGAAAGA -3′. The primers used for B7-H1 (GeneBank: BC113734.1) were 5′- ACAGAGGGCCCGGCTGTTGA -3′ and 5′- AGCGGTACACCCCTGCATCCT -3′. The reaction were conducted by 40 cycles of 95°C 15 s and 60°C 1 min. PCR products for both B7-H1 and 18S were confirmed by sequencing. We used the CT method for calculating: mean relative quantification=2^-meanCT (CTtarget-CTendogenous control)^. B7-H1 levels in the control group were designated as 1.

***Flow cytometry:*** As after transcription, some other reasons, for example post transcriptional, translational mechanisms could also influence gene expression. We used flow cytometry to determine B7-H1 protein. For this, 6×10^5^ HPT-80 cells were cultured in 6-well plates. Cells were treated and cultured in media with different HCMV titers for 24 hours. Cells were harvested using trypsin and cell density was adjusted to 1×10^6^/ml with PBS. Two hundred microliters of cell suspension was incubated in the dark with PE-labeled mouse antihuman B7-H1 monoclonal antibody for 30 min. FACS Calibur (Becton Dickinson) flow cytometry was used for this experiment. Each experiment was repeated at least 3 times. 

For checking the role of different MAPKs, we determined B7-H1 expression by inhibiting different MAPKs. In these studies, stock solutions of MAPK inhibitors were prepared in DMSO. The final concentration of SP600125 (10 μM), U0126 (10 μM), and SB203580 (20 μM) were added to each group and DMSO final concentration never exceeded 0.05%. Different titers of HCMV with medium were added to each group after one hour. The control group was treated with the same amount of DMSO but no MAPK inhibitors. Cells were cultured conventionally for 24 h and the expression of B7-H1 protein was detected by flow cytometry.

***Protein preparation and immunoblotting: ***First, 8×10^5^ HPT-8 cells were cultured in a 100 mm dish and treated as above. The cells were lysed using RIPA buffer [50 mM Tris-HCl (pH 7.4), 1% NP-40, 0.25% Na-deoxycholate, 150 mM NaCl, 1 mM EDTA, 1 mM PMSF, 1 mM Na3VO4, 1 mM NaF, and protease inhibitors (Complete Mini; Roche, Switzerland)]. After sodium dodecyl sulfate–polyacrylamide gel electrophoresis and transferring to a nitrocellulose membrane, the membrane was immunoblotted with anti-human phosphorylated ERK1/2 (P-ERK1/2), P38 or JNK/JNK respectively. The membrane was then incubated with horseradish peroxidase–conjugated secondary antibody, and visualized by enhanced chemiluminescence reaction using SuperSignal West Pico Chemiluminescent Substrate (Thermo Scientific, U.S.). The membranes were stripped and reprobed with antibodies recognizing the corresponding non-phospho proteins.

***Statistical analysis: ***The statistical analysis software package SPSS11.5 was used in this study. Values were means ± SEM. One-way ANOVA was used to analyze data from every group, and the LSD t-test was used to analyze multi-comparison of every group at the same time. *P*-values of less than 0.05 were indicative of statistical significance. All experiments had been repeated for at least 3 times in triplicate.

## RESULTS

***Virulence:*** TCID50 of HCMV AD169 standard strain was measured in HEL cells. According to Reed-Muench calculation, the TCID50 of HCMV AD169 standard strain in this cell came to 10^-^^3^^.29^.

**Fig.1:**
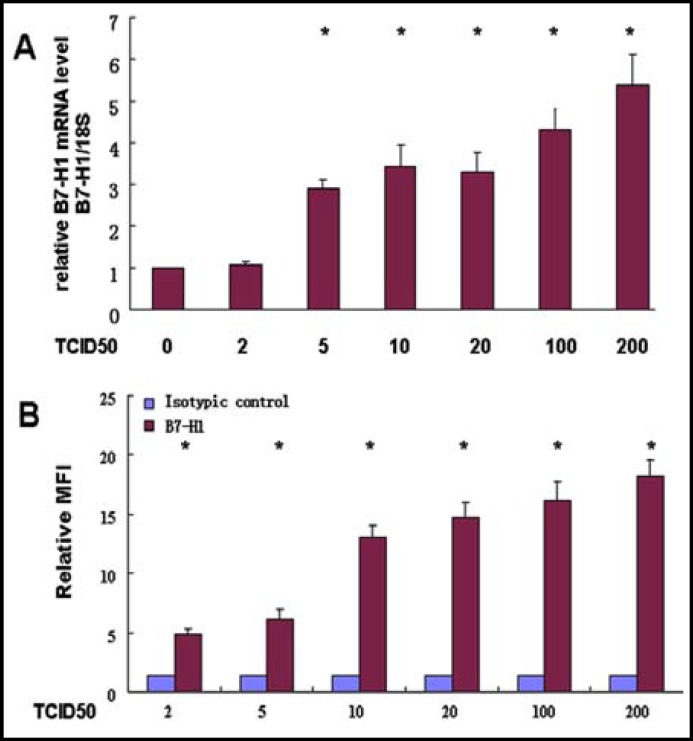
Influence of HCMV on B7-H1 mRNA and protein in HPT-8 cell. A: B7-H1 mRNA was analyzed using real time PCR. B7-H1 levels in the control group were designated as 1 and the relative levels in other groups were calculated accordingly. * indicates a statistically significant difference between indicated group and the viral titer 0 control group (*P*<0.01). B: Influence of HCMV on B7-H1 protein assayed by flow cytometry. * indicates a statistically significant difference between indicated virus treated group and the Isotopic control group (*P*<0.01). Each experiment was repeated at least 3 times

***Influence of HCMV on B7-H1 mRNA:*** Eight hours after HCMV infection, HCMV significantly induced B7-H1 mRNA expression in HPT-8 cells as determined by real time PCR (Fig.1A). There were statistically significant differences between the control and the treated groups with viral titers greater than 5 TCID50 (P<0.01).

***Influence of HCMV on B7-H1 protein:*** Level of B7-H1 protein was measured using flow cytometry (Fig.1B). Changes in B7-H1 expression were assessed by comparison of mean ﬂuorescence intensity (MFI). In all groups, the MFI values were significantly different from the empty control group (*P*<0.05).

**Fig.2:**
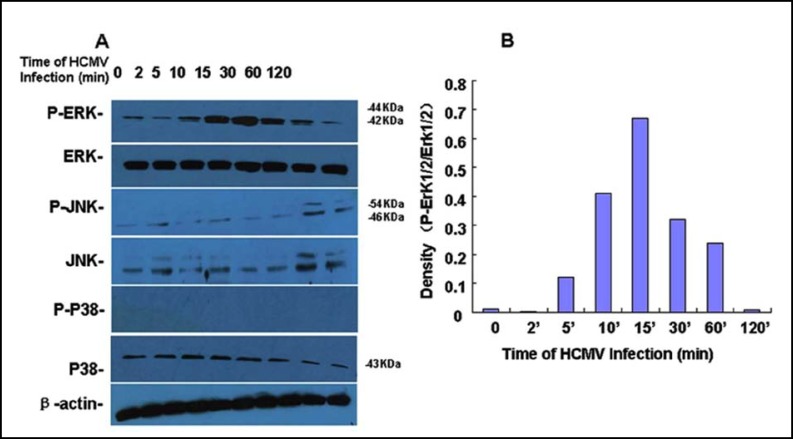
A: Western-blot analysis of the phosphorylation of different MAPKs. After electrophoresis in SDS gel and transferred to a nitrocellulose membrane, the membrane was first immunoblotted with anti-phosphorylated ERK1/2 (P-ERK1/2), P38, or JNK/JNK polyclonal antibody, respectively. After incubation with secondary antibody and visualization of chemiluminescence signal, the membranes were stripped and reprobed with antibodies recognizing the corresponding non-phospho proteins

***Influence of HCMV infection on the phosphorylation of MAPK in HPT-8 cells: ***In order to determine possible mechanisms of HCMV induced B7-H1 expression in HPT-8 cells, we did Western-Blot to check the phosphorylation of MAPKs that could involved in the expression of B7-H1 mRNA. In experiments before, the ability of HCMV to stimulate B7-H1 protein was more obvious when viral titer was 100TCID50. Therefore, an HCMV titer of 100TCID50 was used in all following experiments. Total cellular protein was extracted at different time points after addition of viral solution. ERK/P-ERK, P38/P-P38, and JNK/P-JNK were confirmed by Western blot analysis. ERK, P38/P-P38, and JNK/P-JNK levels did not differ before and after viral infection. However, P-ERK was changed when viral solution was added to the cell. The P-ERK level increased after 5 min and reached its peak at 15 min and gradually returned to its original level at 120 min (Fig.2).

**Fig.3:**
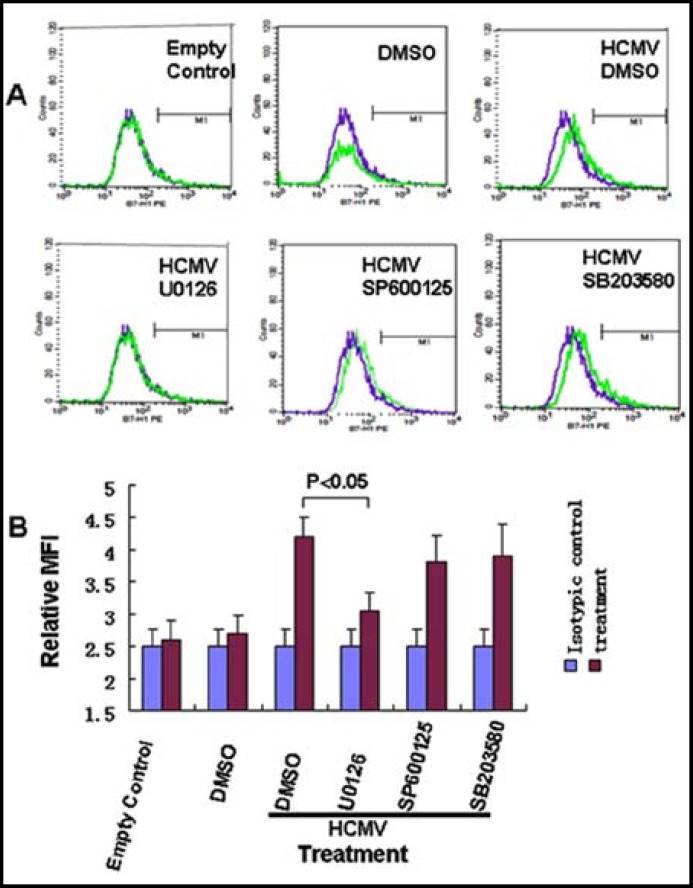
MAPK inhibitors on B7-H1 protein expression caused by HCMV infection, as measured by Flow cytometry. Different inhibitors were added to each group 1 hour before HCMV treatment. The control group was treated with the same amount of DMSO but no MAPK inhibitors. Each experiment was repeated at least 3 times

***Influence of MAPK inhibitors on B7-H1 protein expression caused by HCMV infection: ***Media containing HCMV with different inhibitors of MAPK (JNK inhibitor- SP600125, ERK inhibitor-U0126, P38 inhibitor-SB203580) were added to HPT-8 cells. The B7-H1 protein level was measured using flow cytometry. MFI was used to represent the actual level. No statistically significant differences were observed among the DMSO group, SP600125, and SB203580 (Fig.3). When ERK inhibitor (U0126) was used, however, the concentration of B7-H1 protein decreased significantly (*P*<0.05).

## DISCUSSION

Our results suggested that HCMV infection could cause upregulation B7-H1 mRNA and protein. We also found activation of Erk1/2, one of MAPK pathways, to be involved in this process.

B: Density analysis of P-ERK1/2 / Erk1/2 (Western-Blot result for Erk in graph A).

In the human placenta, B7-H1 protein is expressed selectively on trophoblast cells. This suggests that B7-H1 is important to maternal immunological tolerance of the fetal placenta. Many studies have clearly demonstrated that B7-H1 has dual roles in immunological tolerance: induction and maintenance of peripheral tolerance.^6^ The precise mechanism underlying B7-H1 expression in trophoblast cells is unclear.

Several signal transduction pathways have been reported to participate in the regulation of B7-H1 expression. Crane showed that B7-H1 expression and function in breast and prostate cancer was mediated by the PI3K pathway.^7^ B7-H1 expression was shown to be dependent on the ERK and p38 mitogen-activated protein kinase (MAPK) signaling pathways in colon cancer^8^ **,** in anaplastic large cell lymphoma and Hodgkin lymphoma.^9^ Our study suggests that the ERK 1/2 pathway and resultant increase in B7-H1 transcription are involved in HCMV-induced B7-H1 regulation in trophoblast cells.

 ERK is one important mammalian MAPK pathway. The JNK and p38 MAPK pathways are also important.^10^ HCMV infection has previously been shown to induce activation of ERK1/2 and to be related to viral survival in infected cells.^11^^,^^      1 2^ Using his own studies and those of others, Melnick found that upregulation of ERK phosphorylation is necessary to initial CMV-induced pathogenesis.^      1 3^

HCMV is an important reason for intrauterine infection in perinatal period. In this paper, we have reported the influence of HCMV infection on the expression of B7-H1 and also determined the possible mechanisms underlying. Our result suggested HCMV is a strong inducer of B7-H1 expression in extravillous cytotrophoblast cell line HPT-8, this might explain why HCMV was an important reason for intrauterine infection and might provide useful clues for future anti- HCMV therapies.

## Authors Contribution:

**WRG and JHZ: **Designed the study protocol, conducted most of the study and prepared the primary manuscript. **ZC, LL and HX: **Conducted cell culture, virus titer determination and infection, contributed in the statistical analysis and editing of manuscript. **LHL, XHZ and QST: **Conducted inhibitory study and western-blot. **SHC: **Revised the study design, final revision of manuscript for publication and submitting the paper.

## Notes

***Note: ***W Gong and J Zhao contributed equally to this paper, and both should be considered first author.

## Notes

***Note: ***This work was supported by a grant from China Hubei Provincial Science & Technology Department (Grant No., 2012FFC05601).

## Notes

**Disclosure Statement: **All authors declare no financial interest and no conflicts of interest.
